# An End-to-End Deep Learning Pipeline for Football Activity Recognition Based on Wearable Acceleration Sensors

**DOI:** 10.3390/s22041347

**Published:** 2022-02-10

**Authors:** Rafael Cuperman, Kaspar M. B. Jansen, Michał G. Ciszewski

**Affiliations:** 1 Faculty of Electrical Engineering, Mathematics and Computer Science, Delft University of Technology (TU Delft), Mekelweg 4, 2628 CD Delft, The Netherlands; M.G.Ciszewski@tudelft.nl; 2 Faculty of Industrial Design Engineering, Delft University of Technology (TU Delft), Landbergstraat 15, 2628 CE Delft, The Netherlands

**Keywords:** artificial neural networks, convolutional neural networks, deep learning, football, HAR, human activity recognition, IMU, LSTM, machine learning, soccer

## Abstract

Action statistics in sports, such as the number of sprints and jumps, along with the details of the corresponding locomotor actions, are of high interest to coaches and players, as well as medical staff. Current video-based systems have the disadvantage that they are costly and not easily transportable to new locations. In this study, we investigated the possibility to extract these statistics from acceleration sensor data generated by a previously developed sensor garment. We used deep learning-based models to recognize five football-related activities (jogging, sprinting, passing, shooting and jumping) in an accurate, robust, and fast manner. A combination of convolutional (CNN) layers followed by recurrent (bidirectional) LSTM layers achieved up to 98.3% of accuracy. Our results showed that deep learning models performed better in evaluation time and prediction accuracy than traditional machine learning algorithms. In addition to an increase in accuracy, the proposed deep learning architecture showed to be 2.7 to 3.4 times faster in evaluation time than traditional machine learning methods. This demonstrated that deep learning models are accurate as well as time-efficient and are thus highly suitable for cost-effective, fast, and accurate human activity recognition tasks.

## 1. Introduction

The world of sports has seen a continuous and rapid increase in the usage of technology in both competition and training during the last decades. Specifically in football (soccer), it is nowadays common to see players training and even playing competitive matches wearing vests with GPS trackers below their shirts. These vests can track the players during the whole training or match and give information about their location, distance traveled, speed, power, intensity, and heart rate, among others. These values can be processed to give the players, trainers, and journalists a very detailed analysis of each player’s performance. During the last decade, the scientific community has shown important advancements in Human Activity Recognition (HAR) and, since technology and sports have developed a mutually beneficial relationship, there is a higher demand for systems capable of recognizing specific football-related activities.

If a coach, team, or player has information about which activities the player performs during a match or training, it enables a more detailed analysis of the player’s performance. A more complete assessment of the player’s movements and loads gives the teams the possibility of better training planning, a personalized follow-up to each player, and even a potential way to prevent, treat, and understand injuries. Nowadays, activity classification is done either manually or with the aid of cameras. To this end, it is required to have a large number of high-quality cameras equipped with artificial vision technologies or a considerable number of human labelers. Both of these two options are very expensive and can only be afforded by elite teams. This problem calls for the usage of a low-cost activity recognition system that is also affordable for smaller teams. Sensors are continuously being developed to be smaller, cheaper, and highly accurate (for example, [1]), so their use is a clear solution to this problem. They can be incorporated into the player’s sportswear and provide reliable, real-time measurements of different body parts.

This paper studies the usage of deep learning-based models for football (soccer) activity recognition based on acceleration and angular velocity signals obtained from Inertial Measurement Unit sensors (IMUs). This is done in contrast to traditional machine learning approaches, in which a non-neural network-based model, such as k-nearest neighbors (kNN), decision tree, or support vector machine (SVM), is used to recognize an activity. Traditional methods require a manual process of feature extraction, while the deep learning models take this part into account by themselves. Furthermore, with this work, it is intended to use the raw signals from the IMUs and evaluate how robust the deep models are with respect to the signals acquired on different players. This is why little or no preprocessing will be applied to the sensor outputs, trying to recreate real-life scenarios. Different deep architectures will be proposed for the models and their performance and evaluation time will be examined. Furthermore, a complete training and evaluation pipeline will be designed, in which also the preparation of the training dataset and the strategy for the evaluation phase via a sliding window approach will be taken into account.

The way this paper is structured is as follows. A literature review of the state of the art of methodologies based on sensors in the field of Human Activity Recognition is presented in Section 2. In it, both traditional machine learning and deep learning approaches are shown with their comparison of results, best practices, and challenges. Section 3.1 presents the dataset that was used for training and validating the models. An activity detection algorithm is presented, which is needed for the training phase. In Section 3.2, the training of several deep learning models is thoroughly explained and discussed; and in Section 3.3, the proposed evaluation pipeline is presented, with which the activities present in a given recording can be effectively recognized. The results of the training scenarios and the complete pipeline are shown in Section 4, which are then discussed in Section 5 with the conclusive remarks and future research recommendations.

## 2. Related Work

### 2.1. Machine Learning for Football Activity Recognition

Identifying and recognizing human activities using signals obtained from Inertial Measurement Sensors (IMUs) is an area of machine learning and signal processing that has recently been studied by several authors. Recognition of daily activities, such as walking, climbing stairs, or sitting is especially popular amongst researchers, due to the availability of public domain datasets composed of these types of movements, and the possibility to easily compare the results with previous works [2,3,4,5,6,7,8,9]. On the other hand, studies on recognition of sport-specific activities (such as football, tennis, table tennis, or golf) are less frequent because building these types of datasets is difficult due to the costs involved in resources and time [10,11,12,13,14,15,16]. However, since the nature of the signals is in many cases the same, it is possible to build upon the works of authors who have studied Human Daily Activity Recognition to build accurate and efficient methods designed for an application in a specific sport. The focus of our study is the application in football (soccer).

### 2.2. Traditional Machine Learning Approaches

Human Activity Recognition applications can be developed based on two types of algorithms. The first one will be referred to as traditional machine learning approaches, in which the input features from the signals are manually defined and extracted. This process is not only heavily manual and subjective but is also extremely time-consuming [3]. Common choices of those features are the mean, standard deviation, maximum, minimum, kurtosis, and coefficients of the Fast Fourier Transform [2]. After that, classification is performed using traditional (non-neural network based) algorithms, such as Support Vector Machines (SVM), Decision Trees (DT), k-Nearest Neighbors (kNN), among others.

Several research papers have been written in which Human Activity Recognition is made with these approaches. In all of them, a manually selected set of features is extracted from the signals, usually containing a combination of time- and frequency-domain metrics. The majority of those works focus on daily human activities [3,4,5,6,7,8,9]. Others consider specific sports, such as table tennis [10], tennis [11], skateboarding [12], or volleyball [13]. Among the reviewed articles, ref. [14] studied Human Activity Recognition with application on football. They reported an accuracy of 88.6% on their dataset when using linear SVM. Some years later, ref. [17] developed other algorithms to further explore Football Activity Recognition. In that work, they developed a hierarchical architecture to recognize full-instep kicks, side-foot kicks, or null activities (other movements different from kicks, such as dribbling or running). Their method includes an initial filtering of the signals followed by a peak detection algorithm. The detected peaks are then isolated, a set of several manually selected features are extracted for each peak, and finally, the movement is classified as either a kick or not. They achieved an accuracy of 94% using a Naive Bayes classifier.

Traditional machine learning algorithms require direct human input. Not only for the extraction of relevant features from the input signals but also because in many cases, those features are scale-dependent and sensitive to noise. Hence, performing additional preprocessing is necessary. Various preprocessing techniques are used: filtering [4,10,13,14,15], normalization, standardization [10,13,15], and the usage of norms [4,10,13,14] are common practices. Stroke detection is also frequently used in sports-related applications [10,11,13,15].

### 2.3. Deep Learning Approaches

“The traditional feature engineering methods are becoming more and more incapable” [18]. The second approach for Human Activity Recognition is based on neural networks. One of the most powerful and interesting capabilities of the deep learning approaches is their ability to perform feature extraction without direct human input. This is one of the reasons why recent researchers in areas such as Human Activity Recognition have abandoned traditional approaches in favor of deep learning architectures [19]. An additional important consideration is the general lack of a preprocessing phase of the sensor signals prior to their input into the deep networks. When working with deep learning architectures, the raw signals from the sensors can be used directly. The feature extraction process done by the convolutional and/or recurrent layers can be robust enough to work without any signal preprocessing step.

Moreover, recent works have shown that the use of deep learning approaches for Human Activity Recognition is not only beneficial in terms of feature extraction, but also in achieving high accuracies. The study performed in [2] does an extensive review of different approaches for Human Activity Recognition, and concludes that, on average, traditional machine learning algorithms obtain an accuracy of 83.3%, while systems based on deep learning achieve a much better 94.9%. They also expressed that there are more studies around traditional machine learning algorithms in comparison to deep learning ones, which shows that the use of the latter in Human Activity Recognition tasks is promising, but not yet fully explored.

No relevant scientific publications related to the use of deep learning approaches with sensor data for football activity recognition were found, whereas a large amount of works where these types of algorithms are used for Human Daily Activity Recognition is available. Since the nature of the signals and the ultimate goal of those studies are very similar to the objective of this paper, their methodology and results were considered. Convolutional Neural Networks (CNNs) are very popular because “CNN-based models are able to extract and leverage latent feature representations in time series with high tolerance of time translation; thus, results outperform methods based on hand-crafted features” [16]. In that paper, many different deep architectures were reviewed: from CNN composed of consecutive convolutional layers to more complex and modern possibilities, such as inception CNN and residual CNN. However, “ (...) CNN lacks the capability to capture temporal dependency in time-series sensory data. RNN (Recurrent Neural Network) is designed to model time series data, and it is suitable for discovering relationships in temporal dimension” [20]. This is the reason why the usage of Recurrent Neural Networks was also evaluated by other authors, mainly using Long Short-Term Memory (LSTM) units. A very interesting approach is the combination of CNNs and RNNs to build a larger and, according to the authors of such papers, better performing network. Examples of such architectures are the ones proposed by [18,19,20,21], where usually an RNN (mainly composed of LSTM units) extracts temporal relations of the signals following a feature extraction process made by a CNN.

### 2.4. Background on Deep Learning

Unlike traditional machine learning algorithms, deep learning refers to the use of models based on stacked layers of artificial neural networks. By using multiple layers, it is possible to train the model to progressively extract and learn complex features from the inputs. This has a huge advantage over traditional machine learning algorithms since deep learning models perform both feature extraction and the specific machine learning task. There are many different types of neural networks based on variations of the basic structure of artificial neurons. In particular, this work focuses on two of those types: Convolutional Neural Networks and Recurrent Neural Networks (specifically LSTMs).

#### 2.4.1. Convolutional Neural

Convolutional Neural Networks (CNNs) are a type of deep learning models that were originally designed to tackle artificial vision and image processing problems. Studies on vision and perception of shapes in the human brain showed that the neurons responsible for those tasks have receptive fields. This means that each cell responds to a certain pattern. The combination of these simple patterns generates more complex ones that are furthermore combined so that the brain can finally interpret and understand the image. Convolutional neural networks try to replicate this behavior and have shown impressive results in a huge variety of machine learning applications. Although originally designed for recognizing shapes and figures in images, CNNs can also be used to extract patterns from signals.

The general idea of CNNs is based on two types of operations [22]:

- Convolution
  In a convolutional layer, the input data are convolved with a certain number of kernels or filters. A filter *k* is traversed through all the points of the input image (or signal) and on each location, the convolution between the filter and the overlapping area of the data is calculated. The usage of each filter results in a new convolved image (or signal) called a feature map. The combination of all the *k* filters with different weights then generates a set of *k* feature maps.
- Pooling
  It is a common practice to include pooling layers after convolutional ones. These types of layers condense information from spatially or temporally close points to reduce the data size. In neurology, a receptive field of a neuron is defined as the region in which the presence of a stimulus triggers the response of that particular neuron [23]. Pooling layers introduce the concept of receptive fields into CNNs because they condense information of neighboring points of the convolved data into a single value. Many types of pooling layers can be used, but one of the most common is the max pooling layer. This operation defines a small block (also called a filter) and runs it on top of the input data of the layer. At each point, the maximum of the values contained on the overlapping area of the data and the filter is extracted and the output image (or signal) is built with those maximum values. Pooling layers have the additional property that they reduce the size of the feature maps, which translates into fewer weights to be learned by the model. The outputs of these layers are smaller feature maps, but each element of those maps has information about its neighbors from the previous layer.

#### 2.4.2. Recurrent Neural Networks and LSTM

Recurrent Neural Networks (RNN) are a type of deep learning models that are especially designed to work with data that have an underlying temporal sequence. Because of that, they are typically used for Natural Language Processing and Signal Understanding. With this type of data, it is important to take into account past information since the information is treated as a sequence and what has happened in the past has influence on what will happen in the future. Based on this idea, RNNs have the ability to “remember” prior inputs when generating the output. They produce outputs not only based on the current input vectors, but also on the so-called hidden state vectors that carry information about prior data [22,24].

A major drawback is found when training RNNs due to a problem called “vanishing gradients”. Basic RNNs are unable to remember long-term dependencies because the gradients (how the weights of the neural network change with respect to the loss function that wants to be optimized) that are used to train the model tend to disappear as the input sequence grows in length [25]. When training deep learning models, small gradients are undesirable as the training takes longer and, as a result, becomes less effective.

When working with sequence data such as signals produced by a set of sensors, it is important to have a model able to handle long-term dependencies. This is the reason why more complex RNN cells were developed. Long Short Term Memory (LSTM) cells are one of the most common RNN networks that are used to overcome that problem [24]. They are built upon the basic RNN cells in which prior information is captured in hidden cells and used to generate outputs.

In order to understand how LSTMs work, it is easier to analyze them by parts. One of the most important properties of LSTMs is their capacity to easily propagate information from one cell to another. This is implemented by the cell state $c_{t}$, which can run through the cell with only minor linear modifications. The cell state is the heart of the LSTM and is what carries past information of the input sequence. LSTMs can add or remove information from the cell state by using structures called gates, which are themselves regular feed-forward neural networks operating mainly with an input tensor *x* and a hidden tensor *h*. There are three gates in an LSTM unit:

- Forget gate
  This gate is responsible for deciding what information must be forgotten (removed) from the cell state. To do so, it concatenates the hidden state at time t−1 ($h_{t-1}$) and the current input $x_{t}$ and calculates a value between 0 (forget) and 1 (keep) for each element of the cell state $c_{t-1}$.
- Input gate
  This gate is responsible for deciding what new information will be stored in the cell state and where. It is composed of two parts. The first part calculates candidate values to potentially update the cell state, and the second part decides which parts of the cell state must be updated with those candidate values. This completes the update of $c_{t-1}$ into $c_{t}$ determined by the forget and input gates.
- Output gate
  This gate is responsible for deciding which elements of the cell state will be given as the output of the LSTM unit. Only the desired parts of the cell state are output as the new hidden state values $h_{t}$.

## 3. Materials and Methods

### 3.1. Data and Preparation

#### 3.1.1. Data Collection Procedure

**Figure 1:**
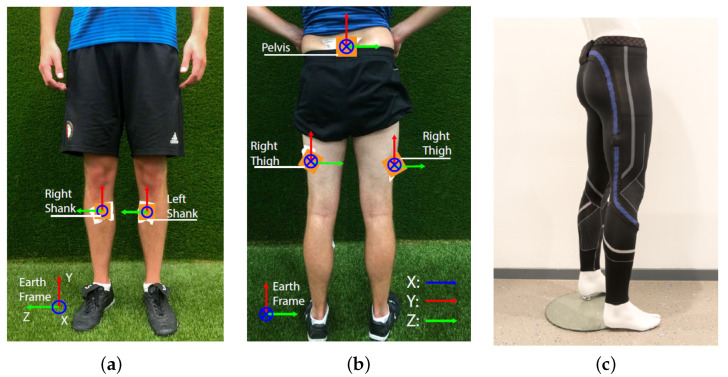
(**a**,**b**): Location of IMUs in experiments. Taken from [28]. (**c**): New sensor tights prototype with fully embedded sensors. Taken from [29].

This work used data acquired by [26] for the training phase and an initial evaluation. For more information and details on the data collection procedures, refer to [26]. The experiments there conducted included 11 male soccer players with 5 IMUs (Ivensense MPU-9150) attached to their bodies in the following locations: pelvis, right thigh, left thigh, right shank, and left shank, as shown in Figure 1. Each one of the IMUs had a tri-axial accelerometer, gyroscope, and magnetometer. The range for the accelerometers was set to ±16 g and for the gyroscopes to $\pm 2000^{\circ }$/s (the magnetometers’ range was not specified. However, they were not used in this work). The sampling frequency of the signals was set to 500 Hz. The sensitivity (with 16 bits) of the accelerometers was set to 2048 LSB/g and of the gyroscopes to 16.4 LSB/(${}^{\circ }$/s). For detailed information of the sensors, refer to [27]. Each one of the participants executed a set of well-specified football-related activities, including passes, shots, jumps, sprints, among others. The experiments were designed in order to simulate an actual football match to have reliable and real movements. The most common football activities were used as the different classes to be recognized: pass, shoot, jump, sprint, and jog.

The use of those 5 IMUs allowed the measurement of the tri-axial accelerations and velocities of the 5 respective body parts related to each one of the activities performed by the subjects. This resulted in several signals representing the movements, each one of them with their manually annotated category (activity). It is important to note that in each experiment, before and after performing an activity, the subject walked or stood still for some time. In order to more effectively train the models, a process of activity isolation was performed in which those low activity intervals were removed prior to training.

#### 3.1.2. Activity Isolation

As explained before, the recordings that were to be used to train the model included activities that were not isolated from surrounding noise and, in order to build a more reliable model, the signals were cleaned so that only the desired activities were present. This follows the logic that the deep learning model learns to extract features by itself. If a lot of irrelevant information would be present in the training phase, it could be possible that the model would learn some features from the low activity patterns and not from the actual activities. Therefore, to make sure that the model learns to recognize accurately the football-related movements, a procedure called activity isolation was developed. It prepared the recordings for the training phase by isolating the important activities from the aforementioned low-activity intervals.

A low-activity measurement is characterized, as its name suggests, by signals with low magnitude and variance which lies in contrast to the behavior of the signal during a high-activity movement. This is especially true when we focus on the acceleration values. When a football player moves from being still or relaxed, the lower limbs accelerate quickly. This is the reason why only the accelerometer signals (and not gyroscope and magnetometer) were used for the activity isolation phase. On the other hand, this big change in acceleration between a low-activity interval and a high-activity one can happen in any of the measured body parts. A standing player can start to run with the right leg while another player can move the left leg first. This is also true with the axes (X, Y, and Z): when jumping the movement is primarily vertical, but when passing we expect the longitudinal component to be more present. In other words, the transition between a low- and a high-activity interval can be detected with any of the body parts and on any axis. This is the reason why the norm of X, Y, and Z axis of each sensor location is used. Each sensor is treated independently and, at the end of the process, they are combined for the final result.

In order to identify when a high activity happens, a baseline value for each signal is obtained: the mean value. When the player is still or walking, the norm of the signal is usually smaller than its mean. However, when the player performs a more intense activity, the norm of the signal presents peaks larger than its mean value. So, the beginning of a high-activity measurement can be found by identifying the moment when the norm of the signal exceeds a threshold based on the mean value. To avoid small meaningless peaks, the algorithm looks for the window of fixed size (based on domain knowledge, we chose 50 timesteps = 0.1 s) in which the norm of the signal exceeds the threshold for each of the timepoints of such window. Similarly, the end of the high-activity interval is identified by placing the window in the opposite direction.

**Figure 2:**
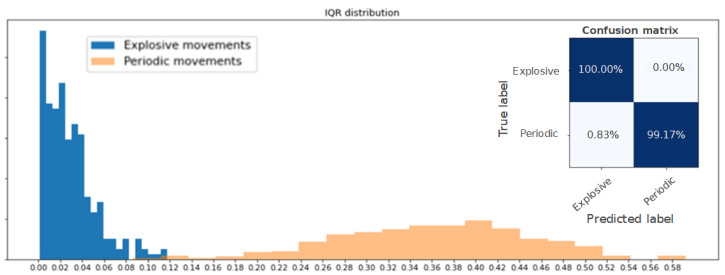
IQR distribution of explosive and periodic movements. On the top right, the confusion matrix of the periodic- vs. explosive-activity classifier based on a threshold of 0.12 on the IQR.

We found that activities such as sprints and jogs required the mean of the signal to be the aforementioned threshold, while movements, such as shots, jumps, and passes, were better isolated when using 1.5 times the mean as the threshold. By understanding the nature of these two groups of movements, the former group was called periodic activities, in which the activity is performed in a periodic manner; and the latter explosive activities, in which the activity is performed only once without repetition. Since explosive activities tend to be shorter and without repetitive patterns, the Interquartile Range (IQR) was proposed as the metric to use to discriminate between both groups of movements. To classify an activity as periodic or explosive, the Euclidean norm of all the accelerometer signals of the recording was taken, then this resulting signal was normalized between 0 and 1, and finally, the IQR was calculated. In Figure 2, the distribution of the IQR values for periodic and explosive activities can be seen. This plot shows that the IQR is a good metric to distinguish between both types of movements if a threshold is chosen. It was defined that if the IQR exceeded 0.12, the recording was considered as a periodic movement or, otherwise, as an explosive movement. This IQR-based classifier showed to have an accuracy of 99.42%, as shown in the confusion matrix on the top right of Figure 2.

**Figure 3:**
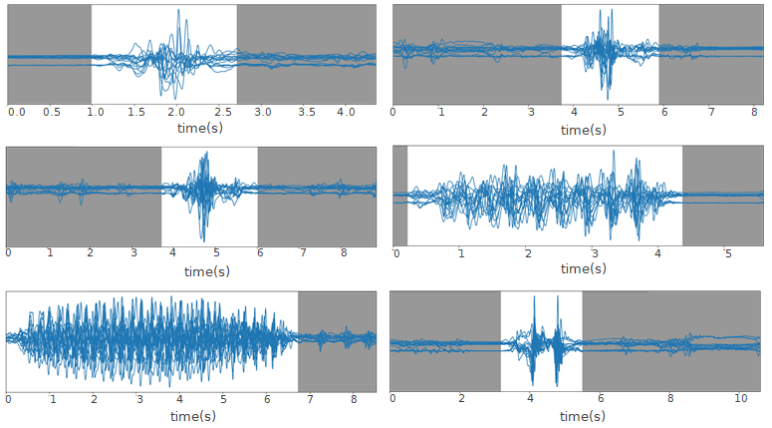
Examples of periods of high activity (white background) obtained after applying Algorithm 1. The examples show accelerometer data related to a shot, pass, pass, jog, sprint, and jump.

Algorithm 1 presents the full procedure of activity isolation. Its application showed to be very effective in isolating high activities from low-activity periods. Some examples of results obtained with this algorithm are shown in Figure 3. In these images, all the signals are superimposed for visualization purposes. The isolated high activities are shown with white background, while the low-activity periods are grayed out.
**Algorithm 1:**Threshold-based activity isolation
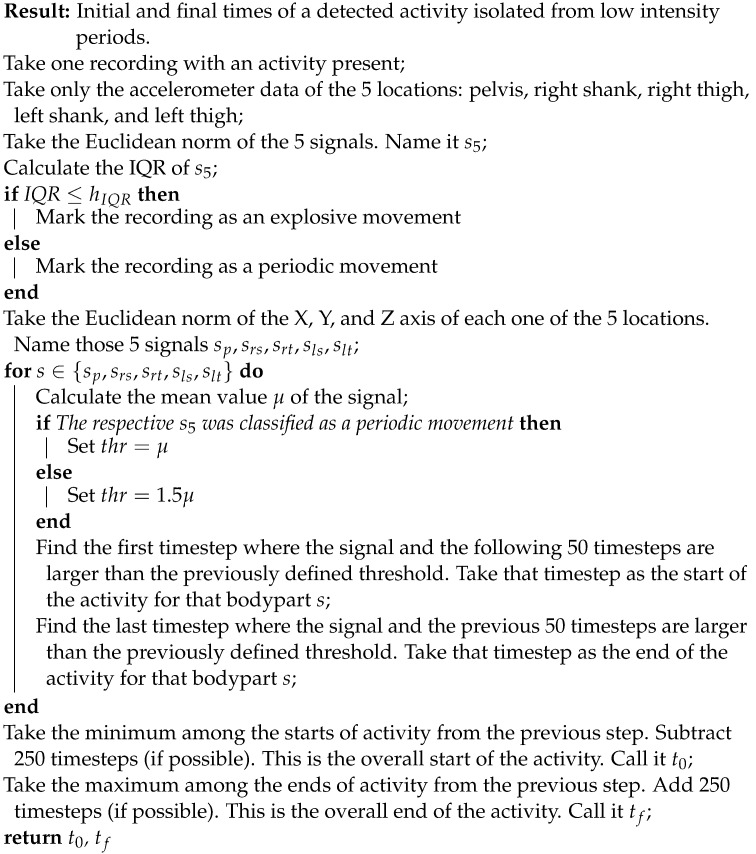


### 3.2. Neural Network Architecture

After the high activities were effectively isolated from low-activity periods, the training and validation datasets were built. The five most common activities in football practice were selected as the classes to be recognized by the deep learning models: shot, pass, jump, jog, and sprint. Since the data were recorded for activities following a well-defined script (e.g., 10 jogs each followed by a shot), the movements were manually labeled. All the examples of those activities were isolated from the original dataset and a window segmentation process was applied to them to extract the training and validation examples. This process consisted of a 1-s-long window traversing through the recordings, extracting, at each time, the respective interval from the original signal. In order to capture temporal dependencies, an overlap of 75% was used when extracting the 1-s-long windows, meaning that every 250 ms of a recording, a new interval of 1 s was extracted.

This set of 1-s-long windows was divided in two via the technique of random subsampling cross-validation: a train dataset, composed of a random 70% set of the samples; and a test dataset, with the remaining 30%. The former was used to train the models and the latter to evaluate them with unseen samples. This train-test split was made by randomly sampling among the recordings of all the subjects. In order to obtain robust results, this procedure was repeated five times (which resulted in five different randomly selected train and test datasets), so that at each time each train and test dataset contained samples from different subjects. The accuracy metrics presented in this paper are the averages of the five repetitions. In other words, the train-test split was performed using a 5-fold random subsampling validation [30]. Additionally, only accelerometer and gyroscope data were used. Magnetometer data were ignored to reduce the dimensionality of the problem. The datasets were balanced via undersampling of the most frequent classes.

As explained before, HAR tasks could benefit from the usage of Convolutional Neural Networks and Recurrent Neural Networks. The former would be responsible for extracting relevant patterns of features from signals and the latter would use those features and give them temporal meaning by understanding the signals as a time series. A combination of both types of layers would be, in theory, very powerful.

**Figure 4:**
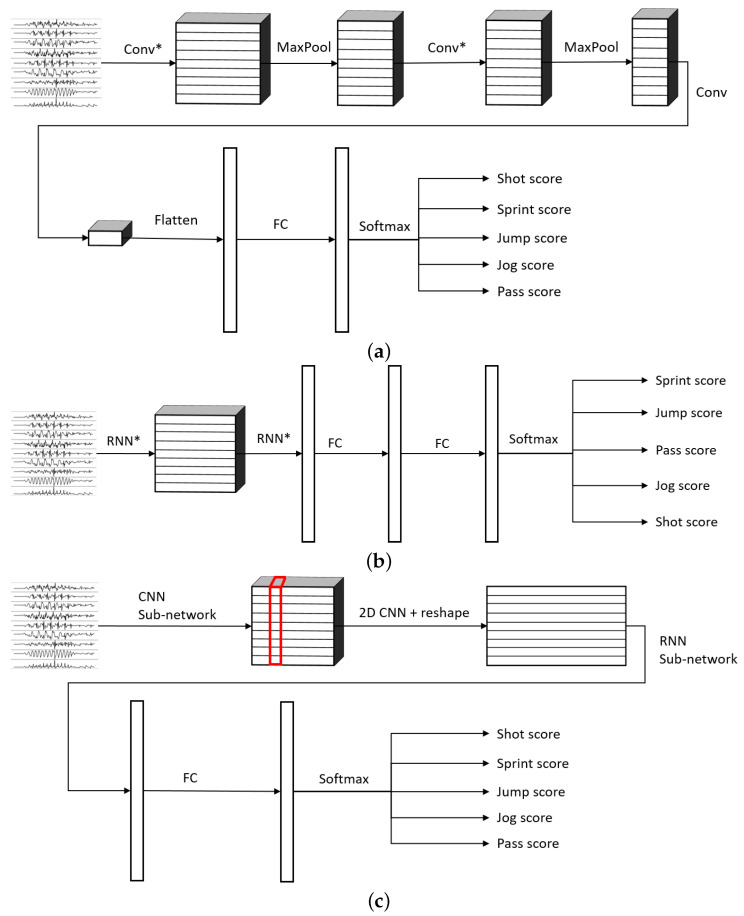
General architectures of the different types of models built. Three types of models were evaluated: using only CNN, only RNN, or a combination of both. The asterisks on the Conv layers mean the usage of a variation of a convolutional layer. The asterisks on the RNN layers represent either LSTMs or bidirectional LSTMs. (FC = Fully Connected Feed Forward Neural Network). (**a**) General architecture for models based on CNNs, (**b**) general architecture for models based on RNNs, (**c**) general architecture for models based on combination of CNNs followed by RNNs.

**Figure 5:**
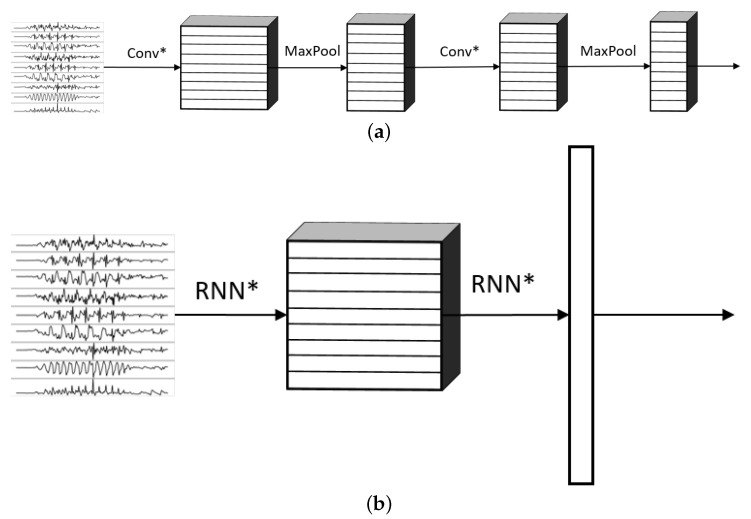
General CNN and RNN sub-networks for the models that combine CNNs and RNNs. The asterisks on the Conv layers mean the usage of different variations of CNNs. The asterisks on the RNN layers represent either LSTMs or bidirectional LSTMs. (FC = Fully Connected Feed Forward Neural Network.) (**a**) General CNN sub-network for the models that combine CNNs and RNNs, (**b**) general RNN sub-network for the models that combine CNNs and RNNs.

The networks proposed follow the same general architectures shown in Figure 4. This paper explored models based solely on CNNs (Figure 4a), RNNs (Figure 4b), and on a combination of CNNs and RNNs (Figure 4c). In Figure 4c, the connections referred as CNN sub-network and RNN sub-network are shown in Figure 5 and they consist of specific types of convolutional and/or recurrent layers that will be explained below.

**Figure 6:**
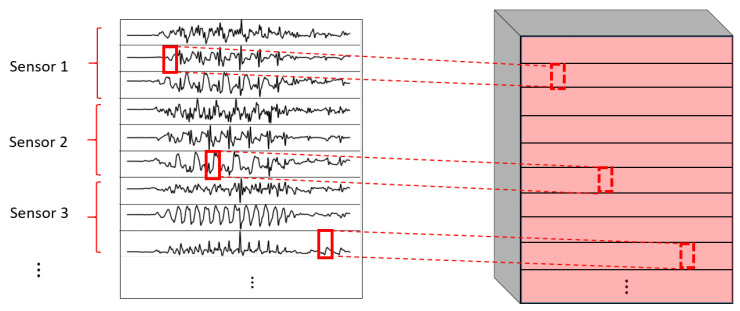
1DCNN weight sharing convolution logic.

**Figure 7:**
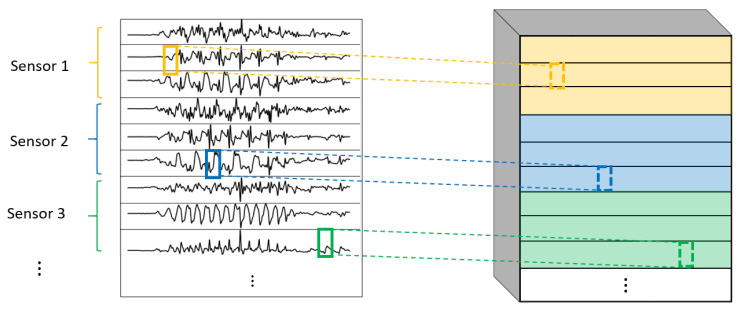
1DCNN per sensor convolution logic.

**Figure 8:**
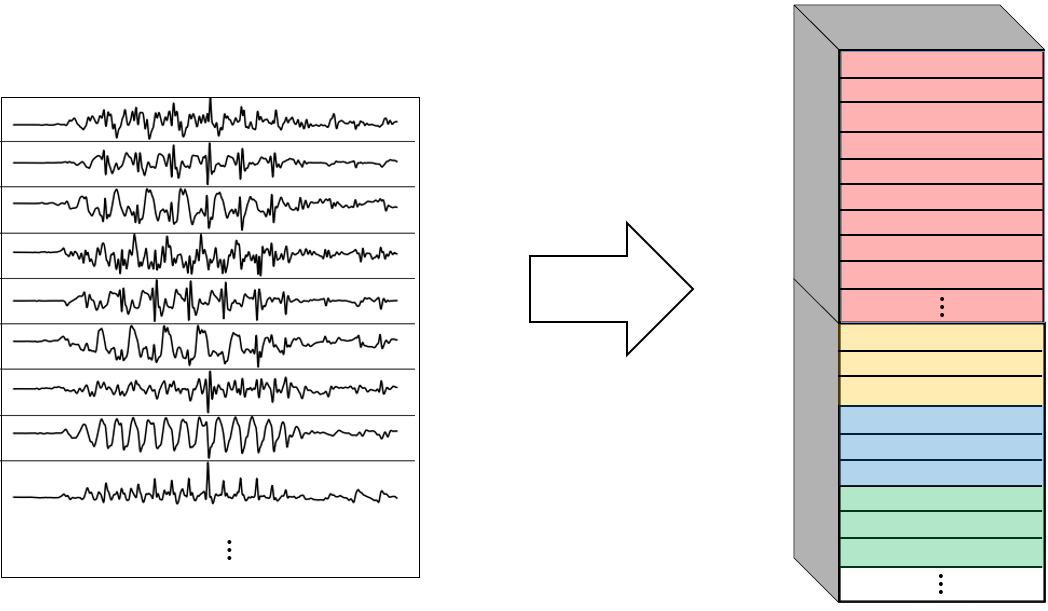
1DCNN combined convolution logic.

**Figure 9:**
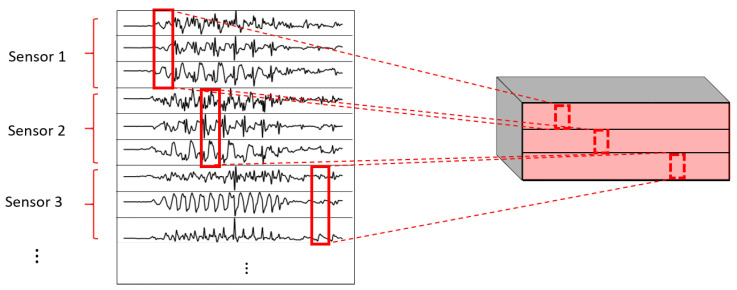
2DCNN weight sharing logic.

**Figure 10:**
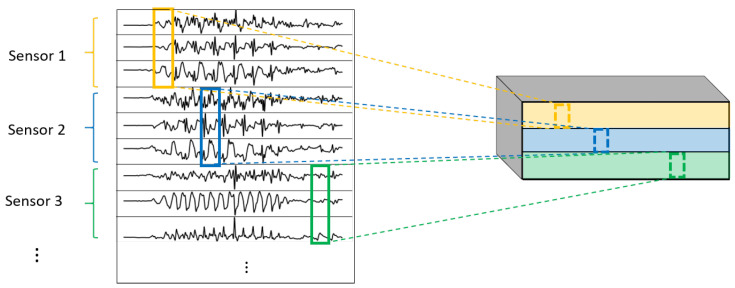
2DCNN per sensor logic.

**Figure 11:**
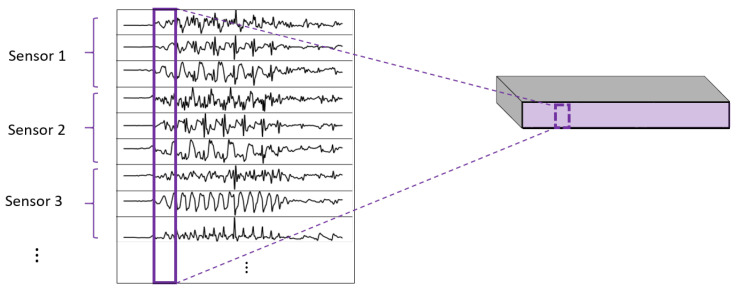
2DCNN all sensors logic.

**Figure 12:**
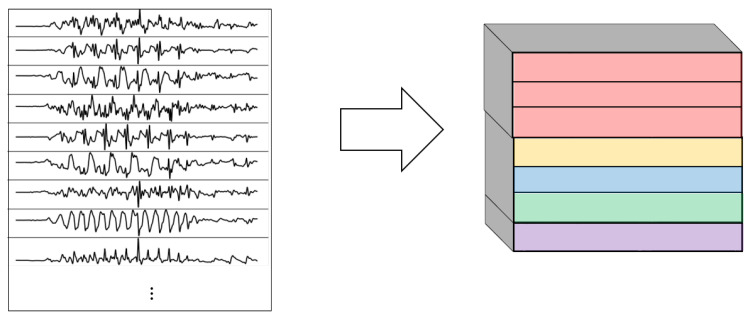
2DCNN combined logic.

One of the most important elements of this paper is the evaluation of different variations of convolutional layers. In Figure 4 and Figure 5, the asterisks in the convolutional parts represent the implementation of the different types of convolutional layers proposed. It is important to note that, even if all the convolutions are theoretically one-dimensional (because the convolution only happens across the temporal dimension), some of them are referred to as one-dimensional and some others as two-dimensional to distinguish among them. By one-dimensional convolution, we refer to convolutions in which the spatial dimension of the filter is 1, so that each signal is processed independently and the filters do not process more than one signal at the same time. By two-dimensional convolutions, we refer to convolutions in which the spatial dimension of the filter is more than 1, so that several signals are convolved simultaneously. The following variations of convolutions were built:

- 1DCNN weight sharing:
  One-dimensional convolutions with the same filters for all the signals. In this type of convolution, filters of size 1×m are used, where *m* is a hyperparameter that determines the timesteps used in the convolution. The 1 implies that each signal is convolved alone. Additionally, weight sharing means that the same filters are used for all the signals. Figure 6 explains this logic. Note that each sensor is composed of three signals (X, Y, and Z axis of the sensor). The convolutions are made for each signal using the same set of *k* filters (represented in red).
- 1DCNN per sensor:
  One-dimensional convolutions with the same filters for all the signals of the same sensor, but different filters for each sensor. In this type of convolution, filters of size 1×m are used, where *m* is a hyperparameter that determines the timesteps used in the convolution. The 1 implies that each signal is convolved alone. However, each sensor has its own set of *k* filters, meaning that the filters are not shared among the sensors. Figure 7 shows this logic. The convolutions are made for each sensor using, for each one of them, a different set of *k* filters (but the same set of filters are used for the three axis of the same sensor). The different sets of filters are represented with different colors in the figure.
- 1DCNN combined:
  Combination of 1DCNN weight sharing and 1DCNN per sensor. Both types of convolutions are performed and their results (feature maps) are concatenated one on top of the other. Figure 8 shows this logic.
- 2DCNN weight sharing:
  Two-dimensional convolutions with the same filters for all the sensors. In this type of convolution, filters of size 3×m are used, where *m* is a hyperparameter that determines the timesteps used in the convolution. The 3 means that the 3 axes of the same sensor are used together in the convolution. In order to convolve each sensor by itself so that specific patterns can be extracted by combinations of the X, Y, and Z signals of the same sensor, a spatial stride of 3 is used. Additionally, weight sharing means that the same filters are used for all the sensors. Figure 9 explains this logic.
- 2DCNN per sensor:
  Two-dimensional convolutions with different filters for each sensor. In this type of convolution, filters of size 3×m are used, where *m* is a hyperparameter that determines the timesteps used in the convolution. The 3 means that the 3 axes of the same sensor are used together in the convolution. In order to convolve each sensor by itself so that specific patterns can be extracted by combinations of the X, Y, and Z signals of the same sensor, a spatial stride of 3 is used. Figure 10 explains this logic. The convolutions are made for each sensor using, for each one of them, a different set of *k* filters. The different sets of filters are represented with different colors in the figure.
- 2DCNN all sensors:
  Two-dimensional convolutions made across all the sensors (thus also signals) at once. In this type of convolution, filters of size NumSensor×m are used, where *m* is a hyperparameter that determines the timesteps used in the convolution. Figure 11 explains this logic.
- 2DCNN combined:
  Combination of 2DCNN weight sharing, 2DCNN per sensor, and 2DCNN all sensors. The three types of convolutions are performed and the resulting feature maps are concatenated one on top of the other. Figure 12 shows this logic.

Specific details of the convolutions used on the different CNN sub-networks are shown in Table 1. There, the following convention is used:

Conv(f, (m, n), s): Convolutional layer with *f* filters of size (m,n) with *s* strides in the vertical (spatial direction) followed with act activation function. In all the cases, a ReLU activation function (f(x)=max(0,x)) is used to include nonlinearities.

MaxP(m, n): Max Pooling layer with filters of size (m,n), as explained in Section 2.4.1.

*d*: Number of spatial dimensions of the output tensor of the previous layer.

The last convolutional layer (which occurs just before the RNN sub-network) is defined as Conv(128, (d, 1), 1). The LSTM and bidirectional LSTM (bLSTM) layers are, in all cases, composed of 128 units. The fully connected layers after the sub-networks are also built with 128 units with ReLU activation function except for the final fully connected layer, which has 5 units (one per each class) and uses a softmax activation function instead. Additionally, to reduce possible overfitting, dropout layers (not shown in the figures) are applied before each fully connected layer.

All of the different architectures were trained using both accelerometer and gyroscope data or only accelerometers to evaluate the influence of gyroscopes on the classification of movements.

To train the models, the ADAM optimization algorithm [31] was used to optimize a categorical cross-entropy loss function. In all cases, the chosen parameters were $\beta_{1}=0.9$, $\beta_{2}=0.999,\epsilon =10^{-8}$. The learning rate (α) was set to decay during the learning phase using a learning rate scheduler. By doing this, the model was able to take large steps towards the optimum during the initial phases of the training, and, as the model approached this point, the steps taken were smaller. This resulted in a better and faster training scheme. The learning rate schedulers for the models were defined as:

- For all the models without any LSTM or bLSTM component, the learning rate was initialized at $10^{-3}$. After every 10 epochs of training, it was reduced to its 75%.
- For all the models with only LSTM or bLSTM (no convolutional part), the learning rate was initialized at $10^{-4}$. After every 10 epochs of training, it was reduced to its 50%.
- For all the models that combined a CNN part with a LSTM or bLSTM part, the learning rate was initialized at $5\cdot 10^{-5}$. After every 10 epochs of training, it was reduced to its 75%.

The training was made with batches of 32 samples for at most 200 epochs. Additionally, an early stopping criterion was defined in order to reduce overfitting and unnecessary training: the validation loss was monitored and if it did not improve (reduce) for 5 consecutive epochs, the training was halted. Each model was trained 5 times using different train-test partitions so that every case used a different subset of data for training and testing. The resulting accuracies of the trainings of each model were averaged to obtain their overall performance.

### 3.3. Postprocessing and Evaluation

Once the models were successfully trained, the next step consisted in using the model to actively recognize and classify the activities present in a recording. A sliding window approach was followed: a window of 1 s (500 timesteps) is swept through the recording and, for each position of the window, a prediction of the activity is made. By doing this, several windows will have periods where no relevant activity is performed because the player is just standing or walking passively. Therefore, the model also needs to be able to recognize these low-activity periods and classify them as such. To allow the model to recognize these low-activity periods, a binary classifier on top of the already trained deep learning-based model was built. That binary classifier was responsible for recognizing whether a window captured a low activity period or not. The *low activity* class could also be included as another class in our models, however, we opted for detecting low activities using a binary classifier before applying the deep model. This is due to the significant differences between low and high activities, which can be exploited to accurately distinguish them.

**Figure 13:**
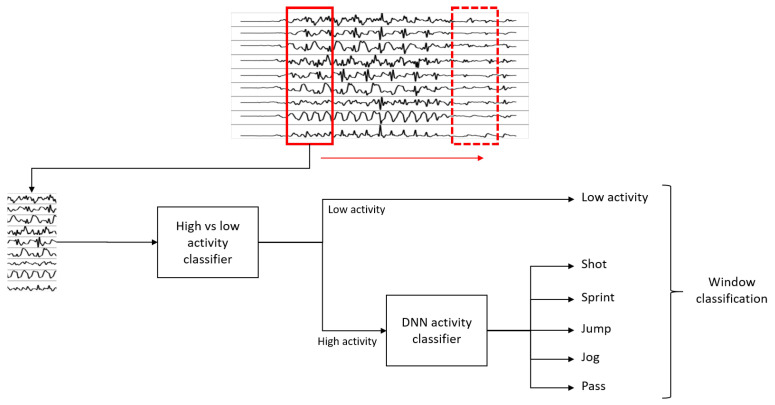
Sliding window evaluation diagram.

The sliding window evaluation phase is shown in Figure 13. A window of the same length as the one used for the training phase (1 s = 500 timesteps) is traversed through the recording, extracting at each time a window of such length. The extracted window is first classified by the binary classifier as either a high or low activity window. If the classification returns *low activity*, the window is understood as such. However, if the binary classifier predicts that the window corresponds to a high activity, then the window is passed through the deep learning model that further classifies the interval as one of the activities: shot, sprint, jump, jog, or pass. This means that, at the end of this process, the window is classified as either shot, sprint, jump, jog, pass, or low activity. This is what we call a prediction. The sliding window moves and a new window is extracted and then classified following the same process.

To build the binary low- vs. high-activity classifier, different metrics were extracted from low- and high-activity windows and evaluated for their discriminant power between both classes. The following values were evaluated for the Euclidean norm of the accelerometer signals:

- Mean;
- Standard deviation (std);
- Coefficient of variation (CV);
- Interquartile range (IQR);
- Range.

The distributions of the metrics for those two groups were examined to identify which one of the statistics would have more discriminatory power between both categories. Additionally, a two-sample Kolmogorov–Smirnov (KS) test was used to evaluate the similarity of these high- and low-activity distributions. The larger the KS value, the more certain we are that both samples come from different distributions. The resultant KS values for the selected metrics are summarized in Table 2.

**Figure 14:**
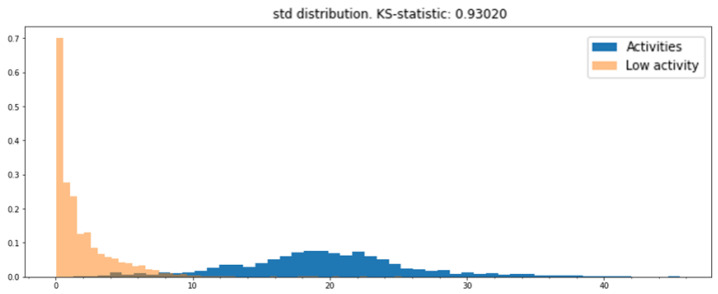
Standard deviation distribution of low- and high-activity windows. Both distributions can be separated using a threshold value.

The standard deviation was chosen as the metric to build the binary classifier since its KS value was the largest. Additionally, the distribution plots of the standard deviation of both categories (high- and low-activity windows) could be separated using a threshold, as seen in Figure 14.

**Figure 15:**
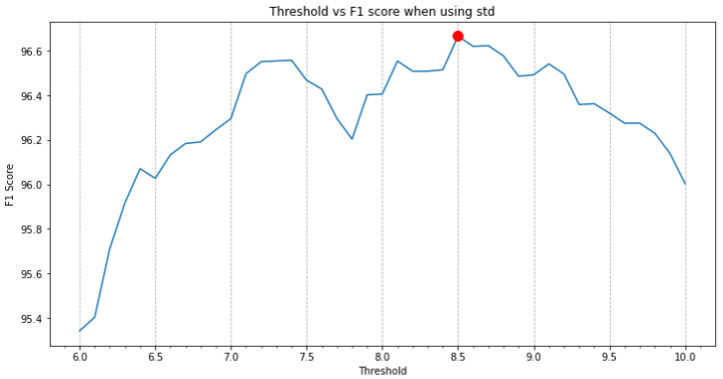
F1 scores for different standard deviation thresholds when classifying high- and low-activity windows. The red dot indicates the chosen threshold.

To define the threshold that should be used for the standard deviation so that the separation between low and high activity was as clean as possible, different values were used to classify a set of new unseen windows with high or low activities: if the standard deviation of the Euclidean norm of the accelerometer signals was larger than a given threshold, the window would be classified as a high activity, and as a low activity otherwise. The F1 score was calculated for each one of the thresholds for the standard deviation and the value where the F1 score was the largest was taken as the optimal threshold. This experiment is depicted in Figure 15 and shows that a threshold of 8.5 gives the best F1 score of 96.67% (and accuracy of 96.56%), which is high enough to consider this binary classifier as good performing.

The complete sliding window evaluation diagram is shown in Figure 13. As it can be seen, the low- vs. high-activity binary classifier lies on top of the deep learning model. We recommend using sliding steps between 10 ms and 100 ms for the windows (windows of 99% and 90% overlap, respectively). It is important to note that, even if the training of the model was made with windows of 75% overlap, this value does not need to be the same as the one chosen for the evaluation phase. The length of the window (1 s = 500 timesteps), on the other hand, must be exactly the same as the one used for the training.

The sliding window approach at the evaluation phase has the issue that there are fewer predictions than timesteps of the recording since the windows are not evaluated at each time point. Furthermore, since there is an overlap between the sliding windows, all the timesteps are evaluated (and therefore predicted) several times by different windows. To solve this problem, the proposed *best-score postprocessing* method postprocesses the predictions so that we can associate a unique activity to each moment of the recording. The proposed method initially assigns all the timesteps of window *i* to the prediction of window *i*. Then, the final prediction for each timestep is the prediction with the largest confidence among the ones of all the windows that contained that particular timestep.

The softmax activation function at the last layer of the neural network was used to calculate the confidences of the predictions. Since the problem is a multiclass classification task, the last layer of the model includes a softmax function. The output of this activation function can be understood as the probability distribution over all the possible categories. The maximum among these probabilities can be taken as the confidence of the predicted class. Therefore, it was defined that a window was considered as “other high activity” if it had confidence lower than a certain threshold (in this work, it was defined as 95%), no matter the prediction that it initially had. With this addition, it was possible to identify movements different from the five predefined ones, such as turns.

**Figure 16:**
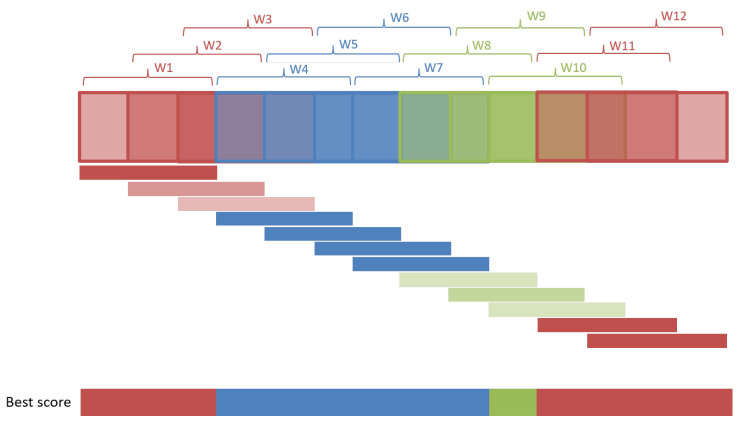
Best-score postprocessing option. The darker the color, the larger the confidence of the prediction.

Figure 16 explains the previously explained process in a graphical, simplified way with a toy example. Suppose that the multicolored horizontal bar on the top of the image represents the recording. That recording is traversed with a sliding window and, for each position of the window, a prediction of the activity is made. Those windows are depicted in the figure as rectangles with thick borders and are named W1,W2,W3,… The prediction made for each window is represented by a color: blue, red, or green. Then, the horizontal bar on the top shows the predictions made by the sliding windows using that color code. That bar is the output of the evaluation pipeline explained in Figure 13. The *best-score postprocessing* method assigns the prediction of the window to all the timesteps contained in that window, as it can be seen with the small horizontal colored bars in the middle of the figure. Each one of those predictions is composed of the recognized activity (red, blue, or green in the figure) and the confidence of the prediction (light to dark tone of the color). Then, as it was mentioned, for each timestep of the recording, the prediction with the largest confidence is taken as the final prediction of the respective timestep. The horizontal bar at the bottom of the figure shows the resulting predictions obtained with the best score postprocessing method. This postprocessing option gives more importance to predictions with high confidence. It has the additional ability of “cleaning” the results of short, isolated, low-score predictions surrounded by predictions with larger confidence.

After postprocessing, an outlier removal procedure is applied to the predictions. Even if the predictions are cleaned with the postprocessing phase, there is still a chance that short peaks of isolated activities remain. An activity performed by a player cannot last less than a certain amount of time in real life. So, if a prediction of a movement lasts less than τ ms, the predicted activity of that interval is replaced with the predicted activity of the next interval that lasts at least τ ms. Good results were obtained when choosing τ to be between 100 and 300 ms.

**Figure 17:**
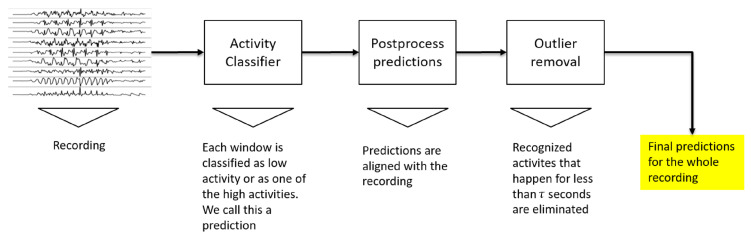
Complete sliding-window evaluation procedure.

The whole sliding-window evaluation process can be summarized with the diagram shown in Figure 17. Each position of the sliding window is classified, then the prediction is postprocessed, and, finally, the outliers are removed. The block called *Activity Classifier* in the diagram is the activity classification process, depicted previously in Figure 13.

## 4. Results

### 4.1. Training Results

As explained before, deep learning models composed of a combination of CNNs and RNNs were proposed, built, and trained to recognize shots, passes, jumps, jogs, and sprints (Figure 4). The training and validation datasets were balanced via undersampling, so that the accuracy could be used as the metric to evaluate the performance of the model. The prediction accuracy is defined as:

$$\mathrm{Accuracy}=\frac{\mathrm{Number}\;\mathrm{of}\;\mathrm{correctly}\;\mathrm{classified}\;\mathrm{samples}}{\mathrm{Number}\;\mathrm{of}\;\mathrm{samples}}$$

When working with deep neural networks, it is a good practice to perform a normalization or standardization of the input data. This has mainly two benefits: the model can be trained faster, and the model can learn to better generalize from the input data [32]. Therefore, the models were trained with and without an initial normalization/scaling of the input signals. Since the signals have both positive and negative values and we wanted to capture information about their positiveness and negativeness, instead of using a min-max normalization, we used a max-abs scaler: we divided by the maximum absolute value of the signal. This process brings all the values to the range [−1,1]. In particular, if the maximum absolute value is found in the positive range, then the values are transformed to the range [−x,1] where x≤1. Similarly, if the maximum absolute value is found in the negative range, the transformed range is [−1,x]. The resulting series has the same shape and structure as the original, but its values lie between −1 and 1 without losing the positive-negative relationship. The max-abs scaling does not shift the data nor destroys the sparsity between positive and negative values. This type of scaling was applied to each one of the signals and the models were trained with these new scaled windows. To have a fair comparison with the non-scaled models, all the models were trained with the same architectures, parameters, and algorithms as the ones previously built. Only the initial learning rate of the models had to be increased 10 times due to much smaller absolute values for the scaled signals. Note that in real-life applications, it would be necessary to perform this scaling with respect to a calibration recording: the player would perform a certain sequence of activities before the competition or training and that recording would be used to scale the subsequent data.

We trained several models based on the proposed convolutional operations and their combination with recurrent layers. The mean and standard deviation of the accuracies of the five trainings of each model can be seen in Table 3 and Table 4. In those tables, a red-to-blue color code was used to visualize the accuracies on the test dataset, where red is worse and blue is better. No color code was given for the train dataset for visualization clarity.

**Figure 18:**
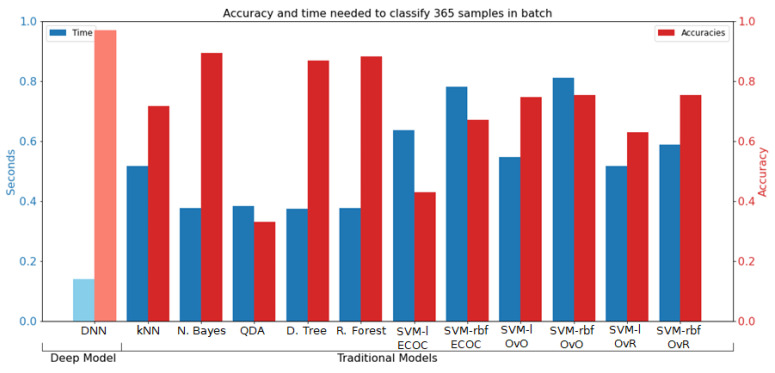
Prediction accuracy and evaluation time for traditional models in comparison to a deep learning-based model (DNN). The blue bars (left vertical axis) represent evaluation time and the red bars (right vertical axis) the prediction accuracy. The names of the models can be found in the main text.

The main goal of this research was not only to have a highly accurate model when recognizing football activities, but it was also desired that those classifications could be made in a short time. To demonstrate the computational efficiency of the proposed deep learning models, traditional machine learning models were trained, and the evaluation times for both types of models were computed. In particular, the following classifiers were built: k-Nearest Neighbors (kNN), Naïve Bayes (NB), Quadratic Discriminant Analysis (QDA), Decision Tree (DT), Random Forest (RF), SVM with linear kernel version ECOC (Error Correcting Output Codes) (SVM-l ECOC), SVM with Gaussian kernel version ECOC (SVM-rbf ECOC), SVM with linear kernel version One-vs-One (SVM-l OvO), SVM with Gaussian kernel version One-vs-One (SVM-rbf OvO), SVM with linear kernel version One-vs-Rest (SVM-l OvR), and SVM with Gaussian kernel version One-vs-Rest (SVM-rbf OvR). For all of them, the following manually selected features were extracted: mean, median, standard deviation, maximum, minimum, skewness, kurtosis, sum of real coefficients of Fast Fourier Transform, and maximum of real coefficients of Fast Fourier Transform. The evaluation times and accuracies of these models in comparison to a particular deep learning-based model (2DCNN per sensor + bLSTM, abbreviated as DNN in the graph) are presented in Figure 18. For the traditional methods, these times include the manual feature extraction process. In general, the deep learning models required about 0.15 s to evaluate 365 samples, while this process took from 0.4 to even 0.8 s with the traditional methods. In addition to this, the best performing deep learning model had the highest accuracy (∼98%), whereas traditional models showed accuracies between 40% and 90%. Deep learning models, thus, perform better on both evaluation time and accuracy.

### 4.2. Complete Pipeline Results

The complete activity recognition pipeline was presented in Figure 13, where a sliding window approach is used to first classify each window of the recording, then postprocess those predictions, and, finally, remove the outliers. Here, some examples of results obtained with the full pipeline are shown. These examples correspond to recordings that were not seen previously by the models, neither during the training nor the validation phase. The figures in this section are composed of four graphs. The one on the top is called the *predictions* and has the predictions obtained by the model (outputs of Figure 17). The second one is called the *postprocessed predictions* and is the result of postprocessing the predictions. The third graph is called the *final predictions* and contains the final predictions after the postprocessed predictions are passed through the outlier removal process (outputs of Figure 17). Finally, the bottom graph is for reference and contains only three of all the sensor signals of the original recording. The horizontal axis (time) is shared among the four graphs. For the top three graphs, the vertical axis corresponds to the predictions made by the model and the orange-black color code represents the confidence of the predictions, where orange means low confidence and black high.

**Figure 19:**
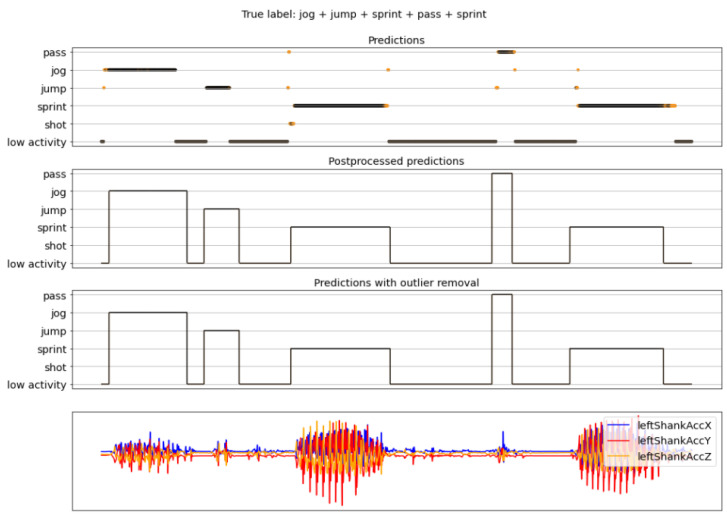
Example 1 of final results. True labels (in order): jog, jump, sprint, pass, and sprint with low activity periods in between each one of them. For the activity prediction plot, the orange-black color code of the dots and lines in the predictions represents the confidence of the predictions, where orange means low confidence and black high.

**Figure 20:**
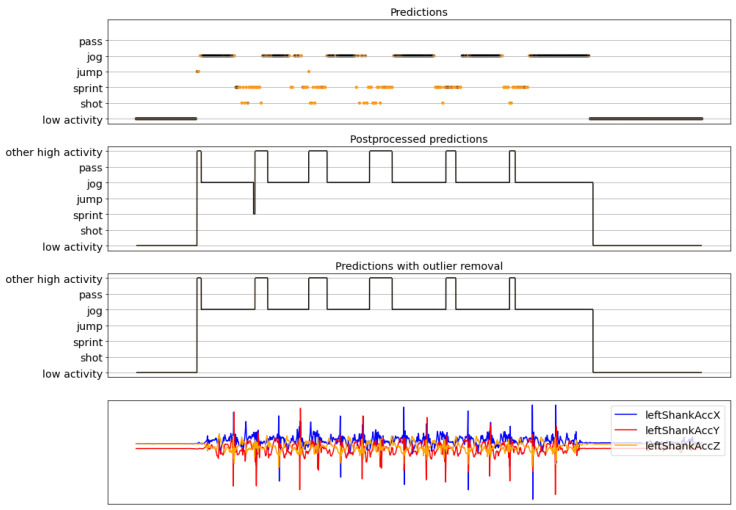
Example 2 of final results. True labels: jog and turn 5 times with a final jog.

**Figure 21:**
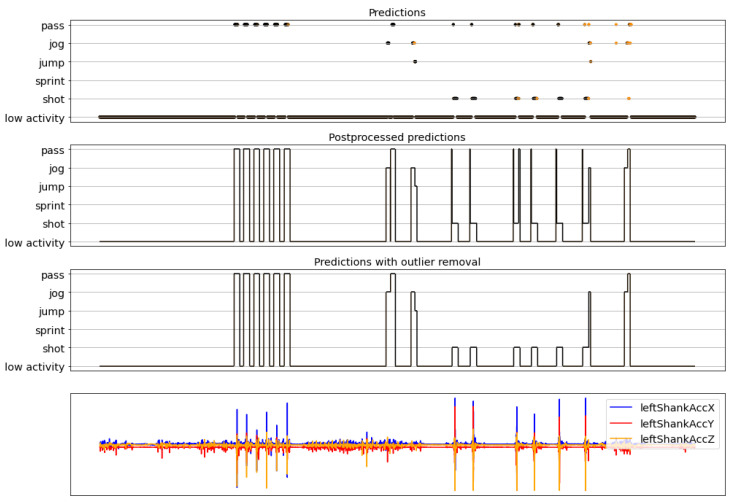
Example 3 of final results. True labels: 6 jumps-passes followed by 6 shots. Fast, unplanned movements are identified before and after the shots.

Figure 19, Figure 20 and Figure 21 present results of the complete pipeline on three different recordings. The true labels of the recordings can be seen in their respective captions.

## 5. Discussion and Conclusions

This paper explored the usage of deep learning models to accurately and rapidly recognize football activities based on IMU measurements from different body parts. The literature review showed that, for Human Activity Recognition, these types of techniques have been taking over the use of traditional machine learning algorithms, such as kNN, decision trees, and SVMs, where a manual process of feature extraction is required. The majority of reviewed articles focused on deep learning models to recognize daily human activities, but this study included explosive and repetitive activities typical of football practice. The possibility of building deep learning-based models on raw, not preprocessed IMU signals was one of the main goals.

A robust and end-to-end pipeline was proposed. It included activity detection and isolation in order to prepare the required datasets, train the models, evaluate a recording via a sliding window approach, and postprocess the results to obtain the final ones. Although the built deep models were trained to recognize the five most common football activities (sprints, passes, shots, jumps, and shots), the proposed methodology can be used to train models to consider additional activities, provided that there are enough training samples of those movements.

A dynamic activity detection algorithm was proposed to isolate activities in recordings from low activity periods. Internally, this algorithm uses a simple classifier that distinguishes between periodic (sprints and jogs) and explosive (shots, passes, and jumps) activities. Then, several deep learning architectures were built, trained, and evaluated in terms of prediction accuracy, overfitting, and evaluation time. Those architectures were based on novel variations of convolutional layers acting across signals, sensors, and/or a combination of them with and without weight sharing. Recurrent layers (LSTMs and bidirectional LSTMs) implemented after the convolutional layers were used to further give temporal meaning to the features previously extracted by the CNNs. The proposed models obtained high accuracies (up to 98.25% in the test set). Compared to traditional machine learning algorithms, deep learning models achieved better accuracies and faster evaluation times, showing that their use is recommended for HAR tasks. The combination of CNNs and bLSTMs was beneficial and achieved better results than the use of only CNNs. Furthermore, models with only bLSTMs did not generate good results, implying that convolutional operations are critical to extract relevant features. Further experimentation is encouraged regarding the training of the models, not only with respect to proposed architectures but also around fine-tuning of architecture and training variables, such as number of layers, number of convolutional filters, type of convolutional operations, learning rate, optimization algorithm, among others.

A sliding window approach for the evaluation phase was implemented following recommendations and best practices found in the literature. With this approach, a complete recording can be evaluated by the models. Postprocessing of the predictions made by the model was necessary, thus the best-score postprocessing method was proposed. This algorithm acts analogous to the non-max suppression algorithm for object detection on computer vision applications. It not only aligns the predictions to the original recording but also filters out short-lived, undesired activities. An outlier removal process was implemented at the end of the pipeline to further refine the final recognized activities from short-lived, unnatural predictions.

Even if the obtained accuracies were high, it is recommended to acquire more data and retrain the models, especially with measurements taken from real matches. The more relevant data that a model can be trained with, the better the learning will be. The literature review showed that HAR is an active research area in the field of deep learning, with several open-source datasets present online ([33,34,35], to name a few). Although the majority of them are about daily human activities, such as walking, sitting, or standing, those large datasets could be used to build a base model, and then use transfer learning to fine-tune it with football data and allow it to recognize specific football activities. The use of transfer learning in deep learning applications has shown to be very effective when building models for tasks that do not have large training datasets and it is expensive or time-consuming to build them. Hence, it is highly recommended to explore this approach to potentially improve the proposed models.

Finally, it is encouraged to use this work as input to future research topics and use-case scenarios such as injury prevention, tracking of activity statistics during competition and training, monitoring physical load, and personalized training. This work is focused on football, but the proposed methodology and pipeline can also be applied to other sports. Recognizing the activities that a player does while playing a sport is just one of the steps that must be done in order to analyze their movements. The recognized activities can be combined with additional data sources such as video recordings and biomechanical analysis to study the prevalence and early detection of injuries. This would help obtain a better understanding of the players’ performance and development.

## Figures and Tables

**Table 1:** Details of layers used on different CNN sub-networks. Recall that the convolutions named *weight sharing* use the same kernels for all the sensors and signals, while the convolutions named *per sensor* use independent kernels for each sensor.

CNN Sub-Network	Layer 1	Layer 2	Layer 3	Layer 4
1DCNN weight sharing	Conv(16, (1, 5), 1)	MaxP(1, 4)	Conv(32, (1, 5), 1)	MaxP(1, 4)
1DCNN per sensor	Conv(16, (1, 5), 1)	MaxP(1, 4)	Conv(32, (1, 5), 1)	MaxP(1, 4)
1DCNN combined	Concatenation of 1DCNN variations			
2DCNN weight sharing	Conv(32, (3, 5), 3)	MaxP(1, 4)	Conv(64, (1, 5), 1)	MaxP(1, 4)
2DCNN per sensor	Conv(32, (3, 5), 1)	MaxP(1, 4)	Conv(64, (1, 5), 1)	MaxP(1, 4)
2DCNN all sensors	Conv(32, (d, 5), 1)	MaxP(1, 4)	Conv(64, (1, 5), 1)	MaxP(1, 4)
2DCNN combined	Concatenation of 2DCNN variations			

**Table 2:** Two-sided KS values for the metric distributions of high- and low-activity window.

Metric	KS Value
Mean	0.9185
Std	0.9302
CV	0.7997
IQR	0.9237
Range	0.9155

**Table 3:** Comparison of mean prediction accuracies between the original unnormalized models and the normalized ones. Five runs. A red-to-blue color code is used to facilitate the visualization of the values on the test dataset, where red is worse and blue is better.

	Acc + Gyro			
	**Normalized**		**Unnormalized**	
	**Train**	**Test**	**Train**	**Test**
**1D CNN weight sharing**	99.93%	97.53%	99.13%	94.36%
**1D CNN per sensor**	99.34%	97.26%	98.35%	93.92%
**1D CNN combined**	99.46%	97.21%	98.66%	93.97%
**2D CNN weight sharing**	99.29%	96.44%	98.80%	93.59%
**2D CNN per sensor**	99.44%	96.55%	99.32%	94.79%
**2D CNN all signals**	99.76%	97.81%	97.58%	92.88%
**2D CNN combined**	99.44%	98.08%	99.34%	95.34%
**1D CNN weight sharing + LSTM**	97.81%	96.11%	99.01%	96.05%
**1D CNN per sensor + LSTM**	99.20%	97.10%	98.31%	95.95%
**1D CNN combined + LSTM**	98.54%	97.04%	98.87%	96.55%
**2D CNN weight sharing + LSTM**	98.54%	96.71%	98.87%	96.27%
**2D CNN per sensor + LSTM**	98.31%	96.71%	99.32%	96.71%
**2D CNN all signals + LSTM**	98.87%	97.32%	98.94%	95.45%
**2D CNN combined + LSTM**	99.08%	97.53%	99.32%	96.71%
**1D CNN weight sharing + bLSTM**	99.39%	98.03%	99.08%	96.55%
**1D CNN per sensor + bLSTM**	99.51%	97.86%	98.66%	96.38%
**1D CNN combined + bLSTM**	99.44%	98.25%	99.22%	96.71%
**2D CNN weight sharing + bLSTM**	99.39%	97.48%	99.20%	96.22%
**2D CNN per sensor + bLSTM**	99.48%	97.04%	98.99%	96.11%
**2D CNN all signals + bLSTM**	99.13%	97.26%	98.45%	94.96%
**2D CNN combined + bLSTM**	99.46%	97.32%	99.29%	96.00%
**LSTM**	63.51%	60.44%	91.48%	77.48%
**bLSTM**	80.49%	76.71%	99.65%	87.07%

**Table 4:** Comparison of standard deviation of the prediction accuracies between the original unnormalized models and the normalized ones. Five runs. A red-to-blue color code is used to facilitate the visualization of the values on the test dataset, where red is worse and blue is better.

	Acc + Gyro			
	**Normalized**		**Unnormalized**	
	**Train**	**Test**	**Train**	**Test**
**1D CNN weight sharing**	0.09%	0.87%	0.81%	1.33%
**1D CNN per sensor**	0.41%	0.74%	1.68%	1.78%
**1D CNN combined**	0.44%	0.70%	0.33%	1.48%
**2D CNN weight sharing**	0.20%	0.81%	0.49%	1.60%
**2D CNN per sensor**	0.29%	0.37%	0.46%	1.42%
**2D CNN all signals**	0.15%	0.67%	0.87%	1.38%
**2D CNN combined**	0.55%	0.35%	0.44%	1.42%
**1D CNN weight sharing + LSTM**	0.79%	0.56%	0.52%	1.21%
**1D CNN per sensor + LSTM**	0.58%	1.44%	1.22%	0.87%
**1D CNN combined + LSTM**	0.54%	0.56%	0.62%	1.60%
**2D CNN weight sharing + LSTM**	0.50%	0.65%	0.92%	1.00%
**2D CNN per sensor + LSTM**	0.76%	1.47%	0.39%	1.31%
**2D CNN all signals + LSTM**	0.75%	0.66%	0.58%	1.02%
**2D CNN combined + LSTM**	0.30%	0.83%	0.37%	1.05%
**1D CNN weight sharing + bLSTM**	0.44%	0.63%	0.34%	0.73%
**1D CNN per sensor + bLSTM**	0.53%	0.68%	0.60%	0.53%
**1D CNN combined + bLSTM**	0.27%	0.66%	0.53%	1.54%
**2D CNN weight sharing + bLSTM**	0.65%	0.97%	0.41%	0.96%
**2D CNN per sensor + bLSTM**	0.35%	1.17%	0.75%	1.10%
**2D CNN all signals + bLSTM**	0.55%	0.62%	0.96%	0.99%
**2D CNN combined + bLSTM**	0.92%	0.82%	0.36%	1.50%
**LSTM**	19.13%	18.19%	2.17%	3.36%
**bLSTM**	24.22%	20.14%	0.24%	2.25%

## Data Availability

Data is not publicly available. Data was obtained from [26] and are available from the authors with the permission of Erik Wilmes upon reasonable request.

## References

[1] Abd M.A., Paul R., Aravelli A., Bai O., Lagos L., Lin M., Engeberg E.D. (2021). Hierarchical Tactile Sensation Integration from Prosthetic Fingertips Enables Multi-Texture Surface Recognition. Sensors.

[2] Slim S., Atia A., Elfattah M., Mostafa M.S.M. (2019). Survey on human activity recognition based on acceleration data. Intl. J. Adv. Comput. Sci. Appl..

[3] Wang L., Liu R. (2020). Human activity recognition based on wearable sensor using hierarchical deep LSTM networks. Circuits Syst. Signal Process..

[4] Adaskevicius R. (2014). Method for recognition of the physical activity of human being using a wearable accelerometer. Elektron. Elektrotechnika.

[5] Mannini A., Sabatini A.M. (2010). Machine learning methods for classifying human physical activity from on-body accelerometers. Sensors.

[6] De Vries S.I., Engels M., Garre F.G. (2011). Identification of children’s activity type with accelerometer-based neural networks. Med. Sci. Sport. Exerc..

[7] Ignatov A. (2018). Real-time human activity recognition from accelerometer data using Convolutional Neural Networks. Appl. Soft Comput..

[8] Ha S., Yun J.M., Choi S. Multi-modal convolutional neural networks for activity recognition. Proceedings of the 2015 IEEE International Conference on Systems, Man, and Cybernetics.

[9] Zebin T., Scully P.J., Ozanyan K.B. Evaluation of supervised classification algorithms for human activity recognition with inertial sensors. Proceedings of the 2017 IEEE SENSORS.

[10] Blank P., Hoßbach J., Schuldhaus D., Eskofier B.M. Sensor-based stroke detection and stroke type classification in table tennis. Proceedings of the 2015 ACM International Symposium on Wearable Computers.

[11] Connaghan D., Kelly P., O’Connor N.E., Gaffney M., Walsh M., O’Mathuna C. Multi-sensor classification of tennis strokes. Proceedings of the SENSORS, 2011 IEEE.

[12] Groh B.H., Kautz T., Schuldhaus D., Eskofier B.M. IMU-based trick classification in skateboarding. Proceedings of the KDD Workshop on Large-Scale Sports Analytics.

[13] Kautz T., Groh B.H., Hannink J., Jensen U., Strubberg H., Eskofier B.M. (2017). Activity recognition in beach volleyball using a Deep Convolutional Neural Network. Data Min. Knowl. Discov..

[14] Schuldhaus D., Zwick C., Körger H., Dorschky E., Kirk R., Eskofier B.M. Inertial sensor-based approach for shot/pass classification during a soccer match. Proceedings of the KDD Workshop on Large-Scale Sports Analytics.

[15] Liu X. (2020). Tennis Stroke Recognition: Stroke Classification Using Inertial Measuring Unit and Machine Learning Algorithm in Tennis. Master’s Thesis.

[16] Jiao L., Bie R., Wu H., Wei Y., Ma J., Umek A., Kos A. (2018). Golf swing classification with multiple deep convolutional neural networks. Int. J. Distrib. Sens. Netw..

[17] Schuldhaus D. (2019). Human Activity Recognition in Daily Life and Sports Using Inertial Sensors.

[18] Xu C., Chai D., He J., Zhang X., Duan S. (2019). InnoHAR: A deep neural network for complex human activity recognition. IEEE Access.

[19] Xia K., Huang J., Wang H. (2020). LSTM-CNN architecture for human activity recognition. IEEE Access.

[20] Lv M., Xu W., Chen T. (2019). A hybrid deep convolutional and recurrent neural network for complex activity recognition using multimodal sensors. Neurocomputing.

[21] Ordóñez F.J., Roggen D. (2016). Deep convolutional and lstm recurrent neural networks for multimodal wearable activity recognition. Sensors.

[22] Goodfellow I., Bengio Y., Courville A. (2016). Deep Learning.

[23] Hubel D.H., Wiesel T.N. (1962). Receptive fields, binocular interaction and functional architecture in the cat’s visual cortex. J. Physiol..

[24] Hochreiter S., Schmidhuber J. (1997). Long short-term memory. Neural Comput..

[25] Pascanu R., Mikolov T., Bengio Y. On the difficulty of training recurrent neural networks. Proceedings of the International Conference on Machine Learning, PMLR.

[26] Wilmes E., De Ruiter C.J., Bastiaansen B.J., Van Zon J.F., Vegter R.J., Brink M.S., Goedhart E.A., Lemmink K.A., Savelsbergh G.J. (2020). Inertial sensor-based motion tracking in football with movement intensity quantification. Sensors.

[27] Invensense (2013). Nine-Axis (Gyro + Accelerometer + Compass) MEMS MotionTracking™ Device.

[28] Wilmes E. (2019). Measuring Changes in Hamstring Contractile Strength and Lower Body Sprinting Kinematics during a Simulated Soccer Match. Master’s Thesis.

[29] Steijlen A., Bastemeijer J., Plaude L., French P., Bossche A., Jansen K. Development of Sensor Tights with Integrated Inertial Measurement Units for Injury Prevention in Football. Proceedings of the 6th International Conference on Design4Health.

[30] Berrar D. (2019). Cross-Validation. Encyclopedia of Bioinformatics and Computational Biology.

[31] Kingma D.P., Ba J. (2014). Adam: A method for stochastic optimization. arXiv.

[32] LeCun Y.A., Bottou L., Orr G.B., Müller K.R. (2012). Efficient backprop. Neural Networks: Tricks of the Trade.

[33] Roggen D., Calatroni A., Rossi M., Holleczek T., Förster K., Tröster G., Lukowicz P., Bannach D., Pirkl G., Ferscha A. Collecting complex activity datasets in highly rich networked sensor environments. Proceedings of the 2010 Seventh International Conference on Networked Sensing Systems (INSS).

[34] Kwapisz J.R., Weiss G.M., Moore S.A. (2011). Activity recognition using cell phone accelerometers. ACM SigKDD Explor. Newsl..

[35] Anguita D., Ghio A., Oneto L., Parra X., Reyes-Ortiz J.L. A public domain dataset for human activity recognition using smartphones. Proceedings of the 21th International European Symposium on Artificial Neural Networks, Computational Intelligence and Machine Learning.

